# Indian consensus statements on the management of small renal masses, non‐muscle invasive bladder cancer and high‐risk/locally advanced prostate cancer

**DOI:** 10.1002/bco2.440

**Published:** 2024-10-03

**Authors:** Simon Hughes, Rajesh Nair, Bhav Radia, Ravimohan S. mavuduru, Prokar Dasgupta, Amit Ghose

**Affiliations:** ^1^ Oncology Department Guy's & St. Thomas' NHS Trust London UK; ^2^ Guy's Cancer Academy Guy's and St Thomas' NHS Foundation Trust London UK; ^3^ King's College London Faculty of Life Sciences and Medicine London UK; ^4^ Urology Department Guy's and St Thomas' NHS Foundation Trust London UK; ^5^ Post Graduate Institute of Medical Education and Research Chandigarh India; ^6^ Apollo Gleneagles Hospital Kolkata India

**Keywords:** consensus, high‐risk prostate cancer, India, non‐muscle invasive bladder cancer, small renal masses

## Abstract

No pan‐India‐specific guidelines exist for the management of urological cancers. Although western guidelines are useful for informing management strategies, they do not account for the nuances of management in the Indian context. A modified Delphi method was used to provide a framework for the systematic development of India‐centric guidelines for the management of three uro‐oncology disease states: small renal masses, non‐muscle invasive bladder cancer and high‐risk/locally advanced prostate cancer.

## 1 INTRODUCTION

Urological cancers encompass a diverse group of malignancies, including prostate, bladder, kidney, and testicular cancers, each requiring tailored management approaches. Although western guidelines offer valuable insights based on extensive research and clinical practice, they may not always align with the unique demographics, healthcare infrastructure and cultural nuances of India. Therefore, having consensus‐based Indian guidelines for managing urological cancers is critical. These guidelines consider the distinct genetic and epidemiological characteristics prevalent in the Indian population, leading to more precise diagnostic and therapeutic approaches. Furthermore, these guidelines consider the country's healthcare disparities and resource limitations, focusing on affordability and accessibility. They also respect cultural attitudes towards cancer and healthcare, fostering better patient–doctor communication and adherence to treatment protocols. By integrating the expertise of Indian oncologists and urologists, these guidelines can provide customised solutions, reducing unnecessary interventions and optimising patient outcomes. Ultimately, consensus‐based Indian guidelines encourage the development of region‐specific clinical trials and research, contributing to the global understanding of urological cancers while offering an approach that resonates with the Indian context.

## 2 METHODS

In 2023, a collaborative group was established between the Urological Society of India: Uro‐oncology Section (USI‐UROONCO), the South Asian Association for Regional Cooperation Association of Urology (SAARC‐AU) and the Uro‐oncology/Guy's Cancer Academy team at Guy's and St. Thomas' NHS Foundation Trust (GCA). The aim was to identify and develop three themes for consensus statements at the Global Uro‐oncology Congress to be held in Kolkata, India.

**FIGURE 1:**
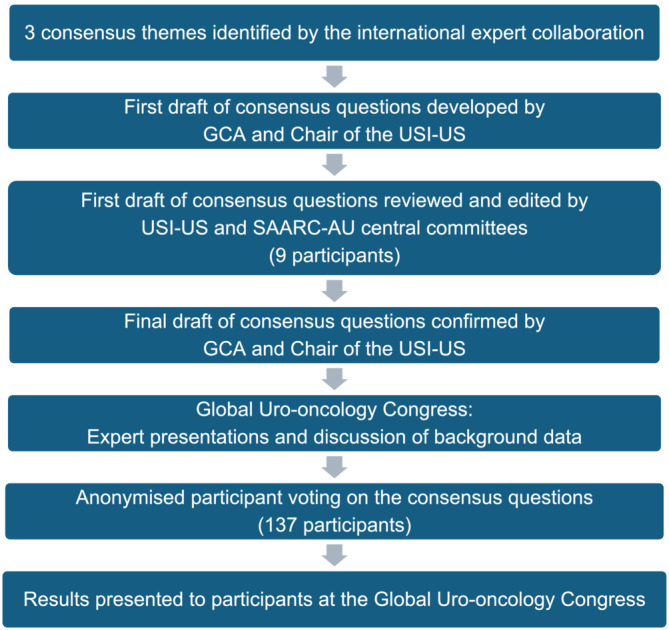
Modified Delphi flow chart.

A modified Delphi technique was followed as outlined in Figure 1. The collaborative group first identified three themes for the consensus statements:

- The management of small renal masses
- The management of non‐muscle invasive bladder cancer
- The management of high‐risk/locally advanced prostate cancer

Within each theme, a first draft of the consensus questions was developed and loaded onto an online portal. Participants from the central committees of the USI‐UROONCO and SAARC‐AU were invited to the platform to give their consent for participation in the consensus process and to review the questions. For each question, the reviewers were asked to comment:

1. Should the question be included using the current wording?
2. Should the question be included using different wording (reviewers were able to suggest a new format)?
3. Should the question be excluded?
4. Are there any further consensus questions that should be included?

The makeup of this review team is shown in Table 1.

**TABLE 1:** Demographics of the central ISA‐US and SAARC‐AU question review team.

Specialty	Postgraduate experience (years)	Clinical work setting	Indian state
Urology	6–20	Government	Uttarakand
Urology	6–20	Government	Maharashtra
Urology	6–20	Government	Gujarat
Urology	6–20	Government	Punjab
Urology	6–20	Trust	Gujarat
Urology	6–20	Private	Uttar Pradesh
Urology	6–20	Private	Telangana
Urology	6–20	Private	West Bengal
Pathology	6–20	Government	Goa

The output from this first round of reviews was reviewed by GCA and the Chair of the USI‐UROONCO to devise a final set of consensus questions to be voted on at the November meeting.

**FIGURE 2:**
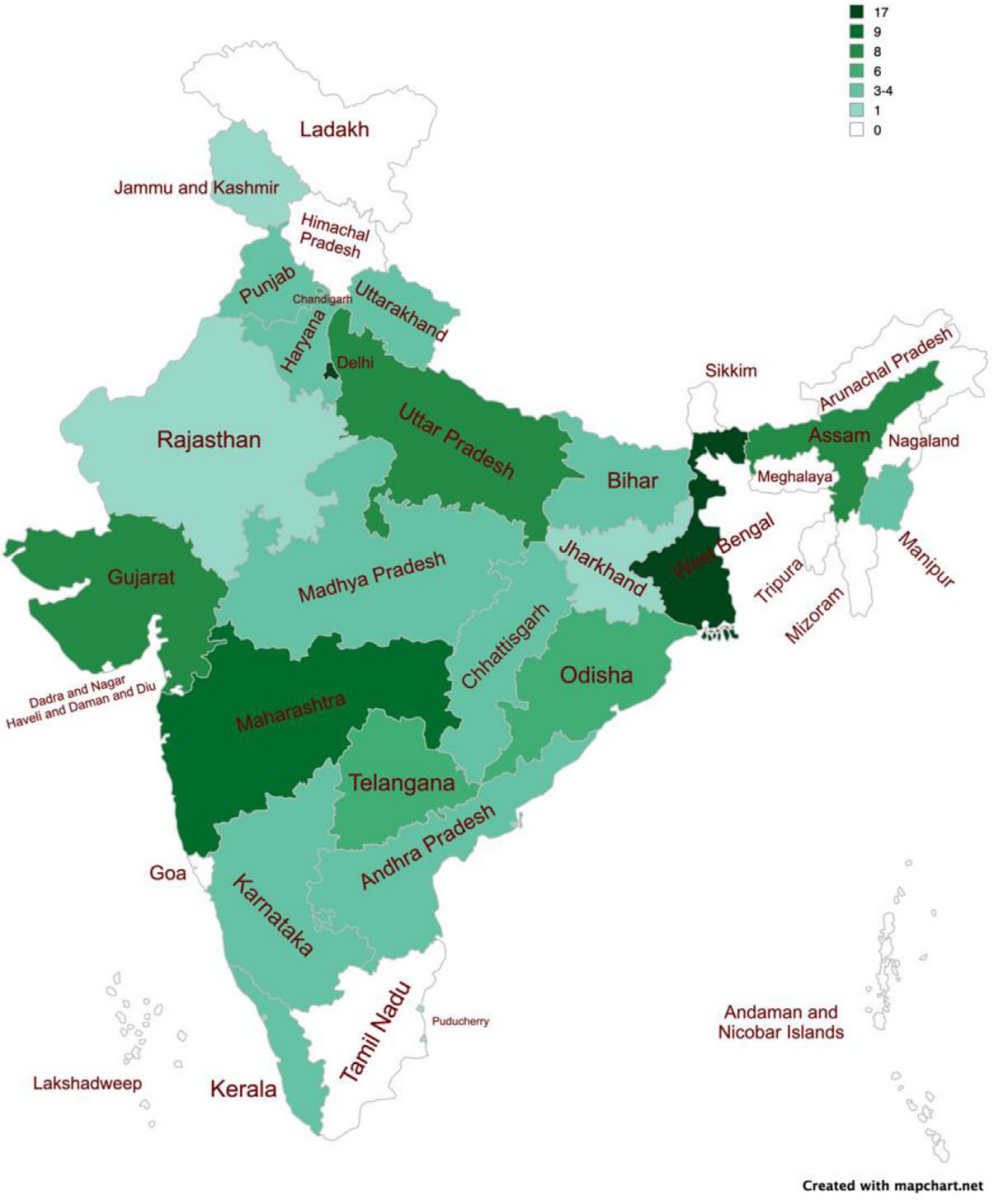
A heat map to display the geographical distribution of participants in the final consensus statement voting.

The Global Uro‐oncology Congress took place on 24th–25th November 2023. All clinical attendees were invited to give their consent to participate in the consensus statement process. The demographics of the participants are shown in Table 2, with a heat map of India displaying where participants were based (Figure 2). Voting was anonymous, using individual voting pads.

**TABLE 2:** Demographics of the consensus audience participants from the Global Uro‐oncology Congress, Kolkata, India 2023.

Specialty					
Urology (%)		Radiation oncology (%)		Total	
139 (96)		6 (4)		145	
Years of postgraduate experience (%)					
1–4	5–9	10–14	15–19	20–24	25+
20 (14)	24 (17)	40 (28)	15 (10)	18 (12)	28 (19)
Clinical work setting (%)					
Government		Charitable		Private	
51		3		91	

**FIGURE 3:**
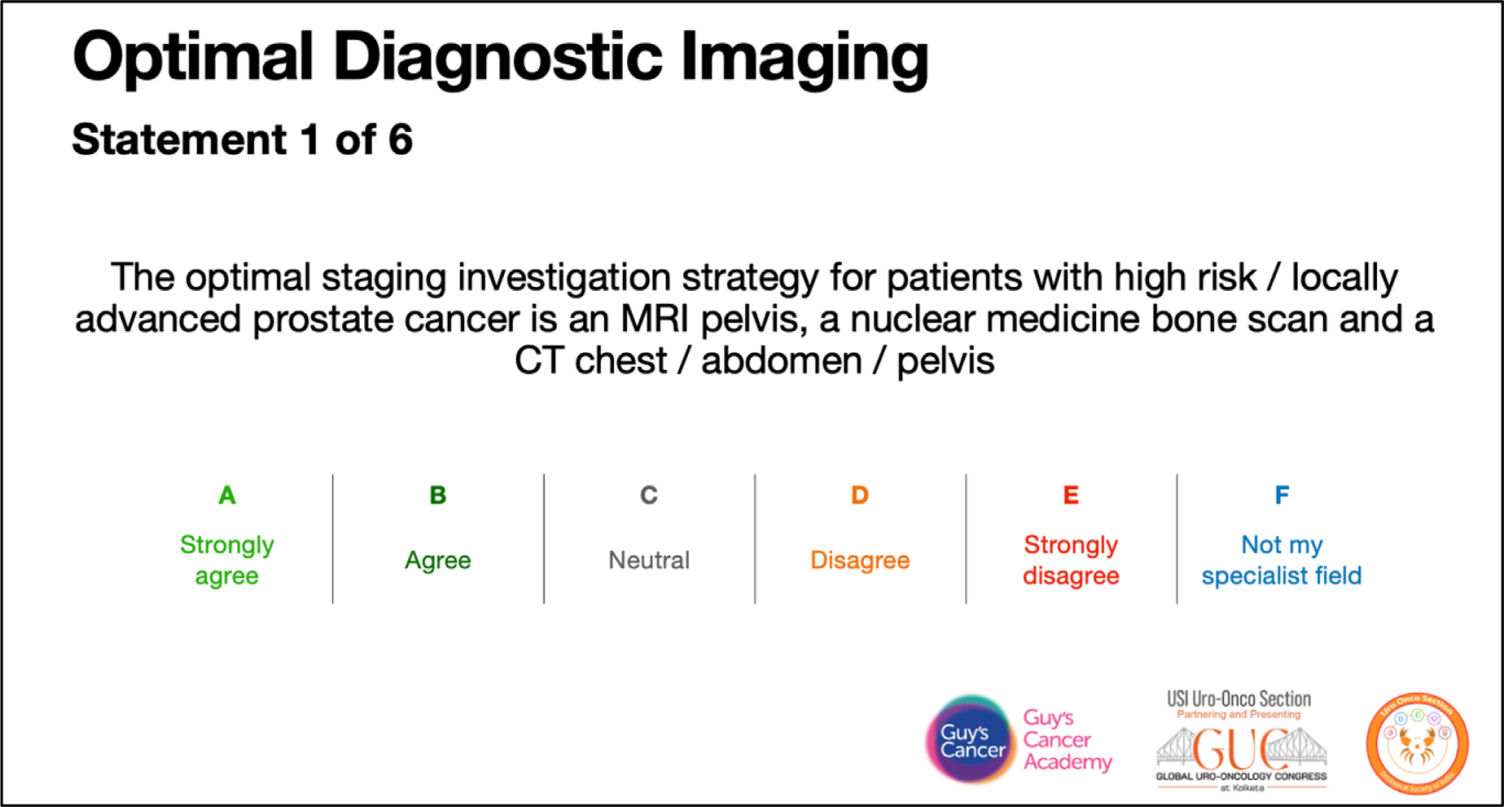
Example of a consensus voting slide (prostate cancer session).

For each theme, the consensus questions were grouped into topics. A clinical expert first presented the global and India‐specific data relevant to each group of questions, before participants were invited to comment and discuss their views. Participants then voted on each question set. For most questions, a Likert voting scale was provided: Strongly Agree/Agree/Neutral/Disagree/Strongly Disagree/Not my Specialist Field (Figure 3); for other questions, discrete options were provided on which to vote.

The following pre‐determined rules were set for a consensus question to be accepted/rejected:

1. All participants were invited to vote on each question—a ‘not my specialist field’ option was provided for those unable to give an expert opinion.
2. ≥75% of votes needed to be in favour (strongly agree/agree) or against (disagree/strongly disagree). For questions where there was a choice of discrete outcomes to vote on, ≥75% of votes needed to be for a specific outcome for it to be accepted.
3. >50% of the total votes cast (including ‘not my specialist field’) also needed to be in favour or against for a question to be accepted/rejected. This was to prevent the acceptance/rejection of statements where only a minority of participants had the relevant expertise to comment.

## 3 RESULTS

The accepted and rejected consensus statements are listed in Tables 3, 4 and 5—by tumour type and sub‐categories. The total number of participants voting on each consensus (excluding those voting ‘not my specialist field’) and the strength of the acceptance/rejection (as a percentage of this number) are also listed. Statements for which a consensus was not reached are shown in Appendices 1–3 (tables 6, 7 and 8). Appendix 4 lists the participants in for the consensus voting.

**TABLE 3:** The consensus results for the management of small renal masses.

Optimal diagnostic imaging			
Statements	Outcome	Number voting	Strength of vote
Patients with a small renal mass should have systemic imaging to complete their staging	Agree	63	81%
The optimal systemic imaging modality for patients with a small renal mass is a CT Chest/Abdomen/Pelvis	Agree	82	88%
The optimal systemic imaging modality for patients with a small renal mass is an FDG PET‐CT	Disagree	86	80%

The role of renal mass biopsy			
Statement	Outcome	Number voting	Strength of vote
Patients with a small renal mass should undergo percutaneous biopsy prior to considering active surveillance	Agree	84	88%
Patients with a small renal mass should undergo percutaneous biopsy prior to considering non‐ionising ablative therapy	Agree	94	95%
Patients with a small renal mass should undergo percutaneous biopsy prior to considering SABR	Agree	89	99%
Patients with a small renal mass should undergo percutaneous biopsy prior to considering partial nephrectomy	Disagree	86	78%
Patients with a small renal mass should undergo percutaneous biopsy prior to considering radical nephrectomy	Disagree	96	84%

The role of active surveillance			
Statement	Outcome	Number voting	Strength of vote
Patients with a small renal mass can be offered active surveillance as a management option	Agree	96	91%
The optimal surveillance imaging modality for patients with a small renal mass is a dedicated kidney CT scan	Agree	100	97%
Patients undergoing active surveillance for a small renal mass should have the frequency of imaging determined by the grade of tumour on biopsy	Agree	86	81%
Patients with a Fuhrman Grade 3 small renal mass on biopsy can be offered active surveillance as a management option	Disagree	89	80%
The optimal surveillance imaging modality for patients with a small renal mass is a kidney ultrasound	Disagree	89	80%

The role of non‐ionising ablative therapy			
Statement	Outcome	Number voting	Strength of vote
Non‐ionising ablative therapy for small renal masses should only be offered in high‐volume centres (a minimum level of expertise needs to be determined)	Agree	78	95%
Patients with a surgically resectable small renal mass, but who decline surgery, can be offered non‐ionising ablative therapy as an alternative management option	Agree	81	97%
Patients with a surgically resectable small renal mass, but who are high risk due to medical comorbidities, can be offered non‐ionising ablative therapy as an alternative management option	Agree	82	97%
Patients with a surgically resectable small renal mass, but who are inoperable due to medical comorbidities, can be offered non‐ionising ablative therapy as an alternative management option	Agree	84	92%
Patients with a surgically resectable small renal mass, but who are at high risk of needing dialysis post‐resection, can be offered non‐ionising ablative therapy as an alternative management option	Agree	72	80%
Radiofrequency ablation is the preferred non‐ionising ablative therapy technique when treating a small renal mass	Agree	66	80%
Patients undergoing non‐ionising ablative therapy for a small renal mass should have a biopsy performed at the time of procedure (if not performed prior)	Agree	76	92%
Patients suitable for cryotherapy ablation of a small renal mass should be offered this percutaneously	Agree	65	92%

The role of SABR (stereotactic ablative body radiotherapy)/SBRT (stereotactic body radiotherapy)			
Statement	Outcome	Number voting	Strength of vote
SABR/SBRT for small renal masses should only be offered in high‐volume centres (a minimum level of expertise needs to be determined)	Agree	68	93%
Patients with a surgically resectable small renal mass, but who decline surgery, can be offered SABR/SBRT as an alternative management option	Agree	88	97%
Patients with a surgically resectable small renal mass, but who are high risk due to medical comorbidities, can be offered SABR/SBRT as an alternative management option	Agree	87	93%
Patients with a surgically resectable small renal mass, but who are inoperable due to medical comorbidities, can be offered SABR/SBRT as an alternative management option	Agree	75	92%
Patients with a surgically resectable small renal mass, but who are at high risk of needing dialysis post‐resection, can be offered SABR/SBRT as an alternative management option	Agree	74	88%

The role of radical nephrectomy			
Statement	Outcome	Number voting	Strength of vote
Patients with a cT1a complex (i.e., potential to be pT3) small renal mass can be offered radical nephrectomy as a management option	Agree	82	96%
Patients with a cT3a small renal mass can be offered radical nephrectomy as a management option	Agree	89	95%
Patients with a central small renal mass close to the hilum can be offered radical nephrectomy as a management option	Agree	88	93%
Patients with a small renal mass close to the collecting system can be offered radical nephrectomy as a management option	Agree	90	91%

The role of nephron‐sparing surgery			
Statement	Outcome	Number voting	Strength of vote
Nephron‐sparing surgery should always be offered where technically possible	Agree	89	98%
Patients with a cT1a small renal mass can be offered nephron‐sparing surgery as a management option	Agree	91	96%
Patients with extensive positive surgical margins post nephron‐sparing surgery for a malignant small renal mass should be offered a completion nephrectomy	Agree	97	88%

Future research themes			
Suggestions	Outcome	Number Voting	Strength of Vote
Genomic profiling of small renal masses undergoing active surveillance	Agree	41	78%
Does treatment of small renal masses improve outcomes?	Agree	62	87%

**TABLE 4:** The consensus results for the management of non‐muscle invasive bladder cancer.

Optimal diagnostic imaging			
Statements	Outcome	Number Voting	Strength of Vote
Optical enhancement techniques (Bluelight‐Hexvix/Narrowband imaging/SPECTRA A/B) should be used to guide a TURBT if recurrent and multifocal	Agree	73	86%
MRI bladder and pinch biopsy can replace TURBT for suspected NMIBC	Disagree	72	82%

Optimal TURBT			
Statement	Outcome	Number voting	Strength of vote
En‐bloc resection of the bladder tumour should be attempted at TURBT	Agree	71	78%
The goal of TURBT should be to identify muscle in the resection specimen	Agree	81	91%
A single dose of intravesical chemotherapy should be delivered following resection of a new NMIBC	Agree	81	95%
Mitomycin C is the preferred intravesical chemotherapy following resection of a new NMIBC	Agree	79	95%
In the context of a normal cystoscopy and CT‐IVU, a positive urine cytology should be investigated with mapping bladder biopsies	Agree	75	95%
In the context of a normal cystoscopy and CT‐IVU, a positive urine cytology should be investigated with bilateral ureteric washings	Agree	83	87%
In the context of a normal cystoscopy and CT‐IVU, a positive urine cytology should be investigated with prostatic urethral biopsies in men	Agree	74	80%
Prostatic urethral biopsies should be taken in all patients suspected of requiring radical cystectomy/cystoprostatectomy prior to consideration of orthotopic neobladder formation	Agree	80	75%
Random cystoscopic biopsies should be undertaken for all patients with a bladder cancer to exclude carcinoma in situ	Disagree	65	81%

First‐line intravesical therapy			
Statement	Outcome	Number voting	Strength of vote
Maintenance intravesical BCG therapy should be continued for 3 years in total in all high‐risk NMIBC patients seeking bladder preservation	Agree	80	86%
If BCG is unavailable, in the presence of significant BCG toxicity, intravesical Gemcitabine chemotherapy can be offered to patients with high‐risk NMIBC seeking bladder preservation	Agree	77	97%
If BCG is unavailable, in the presence of significant BCG toxicity, intravesical Docetaxel chemotherapy can be offered to patients with high‐risk NMIBC seeking bladder preservation	Agree	80	74%
If BCG is unavailable, in the presence of significant BCG toxicity, intravesical Mitomycin‐C Hyperthermia can be offered to patients with high‐risk NMIBC seeking bladder preservation	Agree	74	76%
Fluroquinolone administration should be avoided in patients receiving intra‐vesical BCG‐therapy	Agree	75	76%
Concomitant steroids should be prescribed for patients with BCG‐Cystitis to improve tolerance and reduce symptoms—to improve compliance	Disagree	76	77%

Intravesical therapy for high‐grade recurrence			
Statement	Outcome	Number voting	Strength of vote
Intravesical chemotherapy (Gemcitabine/ Doxorubicin/Docetaxel/Gemcitabine + Docetaxel) can be offered for recurrent high‐grade NMIBC (CIS and T1) post BCG therapy for patients seeking bladder preservation	Agree	72	89%
BCG in combination with intravesical chemotherapy (e.g. mitomycin) can be offered for recurrent high‐grade NMIBC (CIS and T1) post‐BCG therapy for patients seeking bladder preservation	Disagree	69	81%
Radical radiotherapy can be offered for recurrent high‐grade NMIBC (CIS and T1) post‐BCG therapy for patients seeking bladder preservation	Disagree	63	78%

Systemic therapy for high‐grade recurrence			
Statement	Outcome	Number voting	Strength of vote
Systemic immunotherapy (e.g. pembrolizumab) can be offered to patients with persistent/recurrent CIS post‐BCG therapy if seeking bladder preservation/unfit for cystectomy	Agree	49	90%
^a^Systemic immunotherapy (e.g. pembrolizumab) can be offered to patients with persistent/recurrent high‐grade pT1 TCC post BCG therapy if seeking bladder preservation/unfit for cystectomy	Agree	56	95%
Alternative intravesical immunotherapy can be offered to patients with persistent/recurrent CIS post‐BCG therapy if seeking bladder preservation/unfit for cystectomy	Agree	57	98%
Alternative intravesical immunotherapy can be offered to patients with persistent/recurrent CIS post‐BCG therapy if seeking bladder preservation/unfit for cystectomy	Agree	48	98%

Radical cystectomy for high‐grade recurrence			
Statement	Outcome	Number voting	Strength of vote
Radical cystectomy should be offered for persistent CIS post‐induction BCG	Agree	54	100%
Radical cystectomy should be offered for persistent high‐grade pT1 TCC post‐induction BCG	Agree	65	97%

Management of variant histology			
Statement	Outcome	Number voting	Strength of vote
Radical cystectomy should be offered first line to patients with NMIBC and micropapillary changes	Agree	68	90%
Radical cystectomy should be offered first line to patients with NMIBC and sarcomatoid changes	Agree	63	97%
Radical cystectomy should be offered first line to patients with NMIBC and plasmacytoid changes	Agree	62	87%
Neoadjuvant chemotherapy should be offered to patients with NMIBC with small cell neuroendocrine variant histology	Agree	61	87%

Future research themes			
Suggestions	Outcome	Number voting	Strength of vote
Genomic profiling of NMIBC	Agree	37	89%

^a^ Off licence use.

**TABLE 5:** The consensus results for the management of high‐risk/locally advanced prostate cancer.

Optimal diagnostic imaging			
Statements	Outcome	Number voting	Strength of vote
The optimal staging strategy for patients with high‐risk/locally advanced prostate cancer is an MRI pelvis, and nuclear medicine bone scan and a CT chest/abdomen/pelvis	Agree	56	81%
The optimal staging strategy for patients with high‐risk/locally advanced prostate cancer is an MRI pelvis and a PSMA PET‐CT	Agree	69	95%

Transperineal v transrectal biopsy			
Statement	Outcome	Number voting	Strength of vote
The optimal prostate biopsy technique is transrectal ultrasound and transperineal needle biopsy	Agree	82	77%
Whole prostate gland mapping biopsies are required to diagnose prostate cancer and guide management decisions	Agree	83	85%
MRI software‐fusion biopsy is superior to cognitive fusion biopsy to diagnose prostate cancer and guide management decisions	Agree	78	84%
All patients undergoing transrectal needle biopsies of the prostate should receive prophylactic antibiotics	Agree	81	95%
All patients undergoing transperineal biopsies of the prostate should receive prophylactic antibiotics	Agree	80	83%

The role of radical prostatectomy ± pelvic lymph node dissection			
Statement	Outcome	Number voting	Strength of vote
Patients with high‐risk/locally advanced prostate cancer can be considered for a radical prostatectomy	Agree	65	94%
Patients with cT3a prostate cancer can be considered for a radical prostatectomy	Agree	76	94%
Patients with cT3b prostate cancer can be considered for a radical prostatectomy	Agree	73	79%
Patients with low volume cN1 (on PET imaging only) prostate cancer can be considered for a radical prostatectomy	Agree	69	81%
Patients with low volume cN1 (on MRI) prostate cancer can be considered for a radical prostatectomy	Agree	76	87%
Patients with Gleason Grade Group 4 prostate cancer can be considered for a radical prostatectomy	Agree	75	93%
Patients with Gleason Grade Group 5 prostate cancer can be considered for a radical prostatectomy	Agree	71	88%
Patients with a PSA > 20 and prostate cancer can be considered for a radical prostatectomy	Agree	77	90%
Patients with cN0 (on PET imaging) prostate cancer undergoing radical prostatectomy should have a pelvic lymph node dissection	Agree	71	86%
Patients with low volume cN1 (on PET imaging only) prostate cancer undergoing radical prostatectomy should have a pelvic lymph node dissection (this question may be omitted from the final analysis depending on the answer to the previous question on low volume cN1 disease on PET)	Agree	72	96%
Patients with low volume cN1 (on MRI) prostate cancer undergoing radical prostatectomy should have a pelvic lymph node dissection(this question may be omitted from the final analysis depending on the answer to the previous question on low volume cN1 disease) on MRI	Agree	78	96%
Patients with cT4 prostate cancer can be considered for a radical prostatectomy	Disagree	71	80%
Patients with bulky cN1 (on any imaging modality) prostate cancer can be considered for a radical prostatectomy	Disagree	76	78%

The role of radical radiotherapy ± pelvic lymph node radiotherapy ± brachytherapy boost			
Statement	Outcome	Number voting	Strength of vote
Patients with high‐risk/locally advanced cN0 prostate cancer receiving radical hormone radiotherapy should have their prostate, seminal vesicles, and pelvic lymph nodes irradiated?	Agree	49	88%
Patients with high‐risk/locally advanced cN1 prostate cancer receiving radical hormone radiotherapy should have their prostate, seminal vesicles, and pelvic lymph nodes irradiated?	Agree	60	100%
The optimal radiotherapy schedule for high‐risk/locally advanced prostate cancer is: conventional fractionation (2Gy/fraction)	Agree	43	84%
Patients with high‐risk/locally advanced prostate cancer should be considered for ADT + Radical Radiotherapy + a brachytherapy boost?	Agree	47	88%

The role of ADT ± novel androgen receptor‐targeted agents			
Statement	Outcome	Number voting	Strength of vote
Patients receiving radical radiotherapy for very high‐risk/locally advanced prostate cancer should receive 24 months of Abiraterone + Prednisolone (or alternative novel Androgen Receptor Targeted Agent as evidence emerges) in addition to ADT?	Agree	60	95%
What duration of ADT should patients receiving radical radiotherapy for high‐risk/locally advanced prostate cancer receive?	Agree	58	90%
Patients with multiple comorbidities receiving radical radiotherapy for high‐risk/locally advanced prostate cancer should still receive ADT?	Agree	61	85%
Adjuvant ADT can be given to patients with locally advanced prostate cancer post‐radical prostatectomy if pN1 disease found	Agree	56	91%

Prostate cancer survivorship			
Statement	Outcome	Number voting	Strength of vote
Patients receiving ADT should have a structured exercise programme (resistance and cardiovascular)	Agree	36	100%
Patients receiving ADT should receive Vitamin D and Calcium supplementation	Agree	46	100%
Patients receiving ADT should have a baseline DEXA bone density scan	Agree	50	80%
Penile rehabilitation therapy should be offered to all patients undergoing radical prostatectomy	Agree	50	88%
Dedicated pelvic floor physiotherapy should be offered to all patients before and after radical prostatectomy	Agree	48	100%
Patients with erectile dysfunction following radical treatment for high‐risk prostate cancer should be offered specialist advice on management	Agree	50	98%
All patients should have access to a keyworker to coordinate their holistic care needs	Agree	49	95%

Future research themes			
Suggestions	Outcome	Number voting	Strength of vote
The role of trimodality therapy for high‐risk/locally advanced prostate cancer	Agree	36	91%
The impact of PET‐guided management decisions	Agree	42	91%
genomic profiling of high‐risk prostate cancer	Agree	46	96%

## 4 DISCUSSION

This paper represents the first pan‐India consensus statement on the management of Urological cancers. The themes and questions addressed largely concern urological management, as there was insufficient representation from the non‐surgical members of the Uro‐oncology multidisciplinary team to address non‐surgical aspects. Cancer care facilities and resources differ across India, and a strength of the methodology used is that clinical experts from across India, from both government and private institutions, were able to contribute to the statement. Standard practice across India is to adapt international guidelines to the local environment as there is a lack of high‐level evidence generated from the Indian context to guide care. These consensus statements reflect this adaptation of international practice by local clinical experts.

It is interesting to compare these consensus statements with their international counterparts. There are many similarities, but also striking differences based on local practice. For example, both the American Urological Association (AUA) guidelines on small renal masses^1^ and the European Association of Urology (EAU) guidelines on the management of renal cancer^2^ recommend prioritising nephron‐sparing surgery for the management of a cT1a renal mass when intervention is indicated. This approach is mirrored in our consensus with 98% of respondents indicating that nephron‐sparing surgery should always be offered where technically possible for the management of a small renal mass, and 96% indicating that patients with a cTa small renal mass can be offered nephron‐sparing surgery as a management option. When looking at recurrent NMIBC post‐BCG therapy, 90% of our respondents agreed that systemic immunotherapy (e.g., pembrolizumab) can be offered to patients with persistent/recurrent CIS if seeking bladder preservation. This is within its licensed indication with the FDA (US Food and Drug Administration)^3^ based on a phase 2 study,^4^ but a licence has yet to be granted in Europe. However, 95% of our respondents would also offer systemic immunotherapy outside its current licences to patients with persistent/recurrent high‐grade pT1 disease for which it has been shown in trials to have efficacy^5^—indicating a rapid approach to the adoption and extrapolation of data from new studies.

Our consensus statements provide a foundation for establishing India‐centric management guidelines for the management of urological malignancies, against which local practice can be audited. Equally interesting were the statements for which no consensus was reached. There were questions where a consensus was nearly reached, which provide the opportunity for further work using focus groups to determine the underlying reasons for not reaching the threshold for acceptance—for example, could the language in the statements have been made clearer? There were also statements where differing opinions of equal magnitude were recorded. These topics could form the foundation of research using real‐world evidence to explore the factors impacting the divergence of opinion and to look at respective outcomes from centres utilising different management strategies.

Expert re‐review of these statements and the coordination of new consensus statements are future themes for the annual USI‐UROONCO and SAARC‐AU Uro‐oncology congress. Wider participation from the uro‐oncology multidisciplinary team would also allow the application of this process to broader clinical questions within uro‐oncology.

## AUTHOR CONTRIBUTIONS

All authors contributed to the consensus design, data collection, analysis and writing of this paper.

## CONFLICT OF INTEREST STATEMENT

Simon Hughes: Honoraria/travel grants from Astellas, Janssen, Bayer. Prokar Dasgupta: Proximie, MysteryVibe. The other authors declare no conflicts of interest.

## Supporting information


**Appendix S1.** Supporting Information

## References

[1] Campbell SC , Clark PE , Chang SS , et al. Renal mass and localized renal cancer: evaluation, management, and follow up: AUA guideline parts 1 and 2. J Urol. 2021;206:199–208. 34115547 10.1097/JU.0000000000001911

[2] Ljungberg B , Bex A , Albiges L et al. EAU Guidelines on Renal Cell Carcinoma. 2024; https://uroweb.org/guidelines/renal-cell-carcinoma/publications-appendices

[3] FDA approves pembrolizumab for BCG‐unresponsive, high‐risk non‐muscle invasive bladder cancer. 2020; https://www.fda.gov/drugs/resources-information-approved-drugs/fda-approves-pembrolizumab-bcg-unresponsive-high-risk-non-muscle-invasive-bladder-cancer

[4] Balar AV , Kamat AM , Kulkarni GS , et al. Pembrolizumab monotherapy for high‐risk non‐muscle‐invasive bladder cancer unresponsive to BCG (Keynote‐057): a single arm, multicentre, phase 2 trial. Lancet Oncol. 2021;22(7):919–930. 10.1016/S1470-2045(21)00147-9 34051177

[5] Necchi A , Roumiguie M , Kamat A , et al. Pembrolizumab monotherapy for high‐risk non‐muscle‐invasive bladder cancer without carcinoma in situ and unresponsive to BCG (Keynote‐057): a single arm, multicentre, phase 2 trial. Lancet Oncol. 2004;25(6):720–730. 10.1016/S1470-2045(24)00178-5 38740030

