# Supplementary material for: Indian consensus statements on the management of small renal masses, non‐muscle invasive bladder cancer and high‐risk/locally advanced prostate cancer
Source: BJUI Compass. 2024 Oct 3;5(11):1034–43. doi: 10.1002/bco2.440 (PMC11557259; doi:10.1002/bco2.440)
Supplement: Supplementary file 1 — Appendix S1. Supporting Information [file BCO2-5-1034-s001.docx]

**Appendix 1: Small Renal Masses**

Statements that did not meet the threshold for acceptance / rejection are shown in Table 6 (note percentages do not add up to 100% as some participants voted neutral).

**Table 6: Statements that did not reach consensus for Small Renal Masses**

**Optimal diagnostic imaging**

| **Statements** | **Agreed** | **Disagreed** | **Number of votes** |
| --- | --- | --- | --- |
| Patients with a small renal mass should be offered a 99mTc-Sestamibi SPECT/CT to aid local diagnosis and avoid unnecessary surgical excision or percutaneous biopsy | 17% | 69% | 77 |
| Patients with a suspected oncocytoma should be offered a 99mTc-Sestamibi SPECT/CT to aid local diagnosis and avoid unnecessary surgical excision or percutaneous biopsy | 55% | 39% | 88 |

**The role of active surveillance**

| **Statement** | **Agreed** | **Disagreed** | **Number**  **of votes** |
| --- | --- | --- | --- |
| Patients with a complex renal cyst (BOSNIAK-3) can be offered active surveillance as a management option | 47% | 49% | 79 |
| Patients with a small renal mass and a genetic predisposition to Renal Cell Carcinoma (Von Hippel Lindau / Succinate Dehydrogenase Deficiency) can be offered active surveillance as a management option | 52% | 47% | 95 |
| The optimal surveillance imaging modality for patients with a small renal mass is a dedicated kidney MRI scan | 27% | 44% | 97 |

**The role of non-ionising ablative therapy**

| **Statement** | **Agreed** | **Disagreed** | **Number**  **of votes** |
| --- | --- | --- | --- |
| Patients with a surgically resectable small renal mass can be offered non-ionising ablative therapy as an alternative management option | 54% | 36% | 85 |
| Cryotherapy is the preferred non-ionising ablative therapy technique when treating a small renal mass | 58% | 19% | 69 |
| Patients with ipsilateral recurrence of a small renal mass following previous non-ionising ablative therapy can be offered a second non-ionising ablative therapy | 71% | 26% | 80 |
| Patients suitable for cryotherapy ablation of a small renal mass should be offered this laparoscopically | 39% | 40% | 67 |

**The role of SABR (Stereotactic Ablative Body Radiotherapy) / SBRT (Stereotactic Body Radiotherapy)**

| **Statement** | **Agreed** | **Disagreed** | **Number**  **of votes** |
| --- | --- | --- | --- |
| Patients with a surgically resectable small renal mass can be offered SABR/SBRT as an alternative management option | 58% | 35% | 80 |
| Any radiotherapy platform can be used to deliver renal SABR/SBRT | 24% | 67% | 49 |
| What is the optimal non-surgical therapeutic approach for small renal masses in terms of local control (Cryotherapy / Radio-Frequency Ablation / SBRT / Other)? | 39% / 25% / 36% / 0% | | 64 |
| What is the optimal non-surgical therapeutic approach for small renal masses in terms of cost (Cryotherapy / Radio-Frequency Ablation / SBRT / Other)? | 15% / 46% / 35% / 4% | | 52 |
| What is the optimal non-surgical therapeutic approach for small renal masses in terms of toxicity (Cryotherapy / Radio-Frequency Ablation / SBRT / Other)? | 33% / 22% / 43% / 2% | | 63 |

**The role of radical nephrectomy**

| **Statement** | **Agreed** | **Disagreed** | **Number**  **of votes** |
| --- | --- | --- | --- |
| Radical nephrectomy for small renal masses should only be offered in high volume centres (a minimum level of expertise needs to be determined) | 22% | 71% | 82 |
| Patients with a cT1a small renal mass can be offered radical nephrectomy as a management option | 43% | 50% | 82 |

**The role of nephron sparing surgery**

| **Statement** | **Agreed** | **Disagreed** | **Number**  **of votes** |
| --- | --- | --- | --- |
| Nephron sparing surgery for small renal masses should only be offered in high volume centres (a minimum level of expertise needs to be determined) | 58% | 37% | 81 |
| Patients with a cT1a complex (i.e. potential to be pT3) small renal mass can be offered nephron sparing surgery as a management option | 72% | 20% | 94 |
| Patients with a cT3a small renal mass can be offered nephron sparing surgery as a management option | 65% | 33% | 83 |
| Patients undergoing nephron sparing surgery for a small renal mass should have intraoperative frozen section | 28% | 61% | 90 |
| Patients with ipsilateral recurrence of a small renal mass following previous nephron sparing surgery can be offered a second partial nephrectomy | 67% | 26% | 95 |
| Patients with focal positive surgical margins following nephron sparing surgery for a malignant small renal mass should be offered a completion nephrectomy | 28% | 71% | 94 |
| If a patient is undergoing nephron-sparing surgery, and intraoperatively a small thrombus is found in the segmental vein, the surgery should be converted to a radical nephrectomy | 42% | 53% | 89 |

**Future research themes**

| **Suggestions** | **Agreed** | **Disagreed** | **Number**  **of votes** |
| --- | --- | --- | --- |
| Functional imaging to determine the biology of small renal masses | 52% | 17% | 46 |

**Appendix 2: Non Muscle Invasive Bladder Cancer**

Statements that did not meet the threshold for acceptance / rejection are shown in Table 7 (note percentages do not add up to 100% as some participants voted neutral).

**Table 7: Statements that did not reach consensus for Non Muscle Invasive Bladder Cancer**

**Optimal diagnostic imaging**

| **Statements** | **Agreed** | **Disagreed** | **Number of votes** |
| --- | --- | --- | --- |
| Optical Enhancement Techniques (Bluelight - Hexvix, Narrow Band Imaging, SPECTRA A/B) should be used to guide all TURBTs | 40% | 51% | 70 |
| Optical Enhancement Techniques (Bluelight -Hexvix, Narrow Band Imaging, SPECTRA A/B) should be used to guide a TURBT if new and multifocal | 73% | 21% | 70 |
| Patients diagnosed with High grade NMIBC should have a CT chest to complete their staging | 57% | 36% | 75 |
| Patients diagnosed with High grade NMIBC should have an FDG PET-CT to complete their staging | 25% | 73% | 77 |

**Optimal TURBT**

| **Statement** | **Agreed** | **Disagreed** | **Number**  **of votes** |
| --- | --- | --- | --- |
| Fractionated resection techniques should be employed at TURBT | 272% | 14% | 76 |

**First line intravesical therapy**

| **Statements** | **Agreed** | **Disagreed** | **Number of votes** |
| --- | --- | --- | --- |
| Dose reduced maintenance intravesical BCG therapy can replace full dose BCG therapy in patients with high risk NMIBC seeking bladder preservation | 29% | 66% | 80 |
| If BCG is unavailable or in the presence of significant BCG-related toxicity, primary cystectomy should be offered to all patients with high risk NMIBC | 36% | 56% | 80 |
| If BCG is unavailable or in the presence of significant BCG-related toxicity, intravesical Doxorubicin chemotherapy can be offered to patients with high risk NMIBC seeking bladder preservation | 47% | 31% | 72 |
| If BCG is unavailable or in the presence of significant BCG-related toxicity, radical radiotherapy can be offered to patients with high risk NMIBC seeking bladder preservation | 24% | 71% | 78 |
| If BCG is unavailable or in the presence of significant BCG-related toxicity, systemic immunotherapy (pembrolizumab) can be offered to patients with high risk NMIBC seeking bladder preservation | 51% | 37% | 68 |

**Intravesical therapy for high grade recurrence**

| **Statements** | **Agreed** | **Disagreed** | **Number of votes** |
| --- | --- | --- | --- |
| BCG-rechallenge can be offered for persistent CIS post induction BCG for patients seeking bladder preservation | 71% | 29% | 65 |
| BCG-rechallenge can be offered for recurrent high-grade NMIBC (CIS and T1) post BCG therapy for patients seeking bladder preservation | 67% | 31% | 72 |
| BCG in combination with intravesical chemotherapy (e.g. mitomycin) can be offered for persistent CIS post induction BCG for patients seeking bladder preservation | 17% | 72% | 71 |
| Intravesical chemotherapy (Gemcitabine / Doxorubicin / Docetaxel / Gemcitabine + Docetaxel) can be offered for persistent CIS post induction BCG for patients seeking bladder preservation | 71% | 29% | 65 |
| Radical Radiotherapy can be offered for persistent CIS post induction BCG for patients seeking bladder preservation | 21% | 73% | 67 |

**Radical cystectomy for high grade recurrence**

| **Statements** | **Agreed** | **Disagreed** | **Number of votes** |
| --- | --- | --- | --- |
| Minimally invasive (robotic / laparoscopic) radical cystectomy should be the standard surgical approach for cystectomy | 48% | 37% | 65 |
| Extended pelvic lymph node excision should be standard of care during radical cystectomy for NMIBC | 63% | 35% | 63 |
| Frozen section (at urethral / ureteric margins) should be performed in all patients undergoing radical cystectomy for high grade NMIBC | 42% | 52% | 66 |
| All patients who have had a radical cystectomy for NMIBC should have life-long urethral surveillance with urethroscopy or urethral barbotage | 49% | 44% | 63 |
| All patients who have had a radical cystectomy for NMIBC should have life-long upper urinary tract surveillance | 73% | 23% | 64 |
| All female patients undergoing radical cystectomy and urinary diversion for high grade NMIBC should have their uterus and ovaries removed at the same operation | 23% | 73% | 66 |

**Management of variant histology**

| **Statements** | **Agreed** | **Disagreed** | **Number of votes** |
| --- | --- | --- | --- |
| Radical cystectomy should be offered first line to patients with NMIBC and squamous changes | 70% | 25% | 63 |
| Radical cystectomy should be offered first line to patients with NMIBC and neuroendocrine changes | 40% | 60% | 62 |
| Radical cystectomy should be offered first line to patients with NMIBC and glandular changes | 53% | 42% | 60 |
| Radical cystectomy should be offered first line to patients with keratinising squamous metaplasia of the bladder | 49% | 49% | 72 |
| Intravesical BCG therapy scan be offered to all patients with NMIBC and variant histological features (except Small Cell Neuroendocrine) | 40% | 57% | 53 |

**Future research themes**

| **Statements** | **Agreed** | **Disagreed** | **Number of votes** |
| --- | --- | --- | --- |
| Nanofaragene instillation for NMIBC | 68% | 4% | 28 |
| Photodynamic therapy in NMIBC | 61% | 31% | 32 |
| Erdafitinib for NMIBC progressing on BCG | 63% | 15% | 27 |

**Appendix 3: High-Risk / Locally Advanced Prostate Cancer**

Statements that did not meet the threshold for acceptance / rejection are shown in Table 8 (note percentages do not add up to 100% as some participants voted neutral).

**Table 8: Statements that did not reach consensus for High-Risk / Locally Advanced Prostate Cancer**

**Optimal diagnostic imaging**

| **Statements** | **Agreed** | **Disagreed** | **Number of votes** |
| --- | --- | --- | --- |
| The optimal staging investigation strategy for patients with high risk / locally advanced prostate cancer is an MRI pelvis, and a whole-body DW MRI scan | 24% | 66% | 59 |
| In patients with Gleason Grade Group 5 disease or neuroendocrine differentiation, dual PET tracer imaging with FDG and PSMA should be considered | 73% | 23% | 71 |
| What is the optimal PET tracer for staging high risk / locally advanced prostate cancer (F18 PMSA / G68 PSMA / Choline / Other)? | 28% / 68% / 2% / 2% | | 57 |
| Bi parametric MRI is an acceptable alternative to Multiparametric MRI for the local staging of high risk / locally advanced prostate cancer | 60% | 73% | 73 |

**Transperineal v transrectal biopsy**

| **Statements** | **Agreed** | **Disagreed** | **Number of votes** |
| --- | --- | --- | --- |
| The optimal prostate biopsy technique is transrectal ultrasound and transrectal needle biopsy | 70% | 25% | 67 |
| Prostate target lesion biopsy alone is sufficient to diagnose prostate cancer and guide management decisions | 24% | 69% | 83 |

**The role of radical radiotherapy +/- pelvic lymph node radiotherapy +/- brachytherapy boost**

| **Statements** | **Agreed** | **Disagreed** | **Number of votes** |
| --- | --- | --- | --- |
| The optimal radiotherapy schedule for high risk / locally advanced prostate cancer is: moderate hypofractionation (2.2-4Gy/fraction) | 74% | 17% | 23 |
| *The optimal radiotherapy schedule for high risk / locally advanced prostate cancer is: extreme hypofractionation | 82% | 18% | 22 |

* <50% of total votes cast (including “not my specialist field”)

**The role of ADT +/- novel androgen receptor targeted agents**

| **Statements** | **Agreed** | **Disagreed** | **Number of votes** |
| --- | --- | --- | --- |
| Patients undergoing a radical prostatectomy for very high-risk / locally advanced prostate cancer should also receive 24 months of Abiraterone + Prednisolone (or alternative ARTA) in addition to ADT (off license)? | 62% | 35% | 55 |
| Patients undergoing a radical prostatectomy for very high-risk / locally advanced prostate cancer should also receive 24 months of Abiraterone + Prednisolone (or alternative ARTA) in addition to ADT (off license)? | 62% | 28% | 53 |

**Prostate cancer survivorship**

| **Statements** | **Agreed** | **Disagreed** | **Number of votes** |
| --- | --- | --- | --- |
| Patients should be offered duloxetine as first line medical therapy for stress urinary incontinence post prostatectomy | 47% | 47% | 49 |
| Patients with urinary incontinence post radical prostatectomy for high risk prostate cancer should be considered for a urinary sphincter | 60% | 34% | 47 |

**Future research themes**

| **Statements** | **Agreed** | **Disagreed** | **Number of votes** |
| --- | --- | --- | --- |
| Extreme hypofractionation when delivering prostate + Pelvic lymph node irradiation | 72% | 24% | 25 |

**Appendix 4: Consensus Participants**

Dr. Rajesh Kumar Reddy Adapala

Dr. Sanjai Addla

Dr. Anshuman Agarwal

Dr. Mayank Mohan Agarwal

Dr. Puneet Ahluwalia

Dr. Jamal Akhtar

Dr. Madhur Anand

Dr. Haris Ansar

Dr. Shrikanth Atluri

Dr. Brojen Barman

Dr. Sasanka Kumar Barua

Dr. Trinanjan Basu

Dr. Atul Batra

Dr. Vidhur Bhalla

Dr. C Dev Krishna Bhanathi

Dr. Deepak Prakash Bhirud

Dr. Girdhar S Bora

Dr. Sabyasachi Bose

Dr. Samit Chaturvedi

Dr. Percy Chibber

Dr. Gautam Ram Chowdhury

Dr. Manoj K Das

Dr. Saurabh Kr Das

Dr. Animesh Kumar Das

Dr. Nabajeet Das

Dr. P Dasgupta

Dr. Hiranya Deka

Dr. Mahesh Desai

Dr. Preetham Dev

Dr. P N Dogra

Dr. Anil Elhence

Dr. Himesh Gandhi

Dr. Arvind Ganpule

Dr. Thirumalai Gansan

Dr. Harshit Garg

Dr. Amit Ghose

Dr. J Ghose

Dr. Bastab Ghosh

Dr. Nirmalya Ghosh

Dr. Syer Mohammed Ghouse

Dr. Amit Goel

Dr. Ganesh GopalaKrishnanan

Dr. Madhur P Gopalakrishnanan

Dr. Rajesh Gulia

Dr. Anup Kumar Gupta

Dr. Narmada Prasad Gupta

Dr. Piyush Gupta

Dr. Rahul Gupta

Dr. S Gupta

Dr. Khurshid A Guru

Dr. Nirmal T J

Dr. Arshad Jamal

Dr. R Jane

Dr. Tarun Jindal

Dr. Anantha K

Dr. Nagaraj H K

Dr. Shitangsu Kakoti

Dr. D Karmakar

Dr. Nitin Kekre

Dr. M V Khochikar

Dr. Somarendra Khumukcham

Dr. Rajesh Kukreja

Dr. Ajay Kumar

Dr. Amit Kumar

Dr. Anant Kumar

Dr. Nand Kumar

Dr. Prabhat Kumar

Dr. Sanjeev Kumar

Dr. Anupam Lal

Dr. Jivasankar M

Dr. Sanjay Kumar Mahapatra

Dr. C Malikarjuna

Dr. Suman Mallick

Dr. Ashwin Mallya

Dr. Kim J Mammen

Dr. Khatem Matar

Dr. Ravimohan S Mavuduru

Dr. Alan Mc Neil

Dr. Mehul

Dr. Uttam Mete

Dr. Presenne Kumar Mishra

Dr. Sourav Kumar Mishra

Dr. Nagendra Nath Mishra

Dr. Vishal Mishra

Dr. Ankur Mittal

Dr. V Chandra Mohan

Dr. Surendra Mohan

Dr. Indranil Mullick

Dr. Balagopal Nair

Dr. Tushar A Narain

Dr. Akshay Nathani

Dr. Rohan Nautugal

Dr. Brusabhanu Nayak

Dr. N P Padmakar

Dr. Vikas Kr Panwar

Dr. Deval Parikh

Dr. Nitesh Patidar

Dr. Sujata Patwardhan

Dr. Sayan Paul

Dr. Aditya Pradhan

Dr. Ginil K Puleri

Dr. Raghuram

Dr. T Ramamohan

Dr. Rohit Ranjan

Dr. Debashis Routray

Dr. Sreerag K S

Dr. R B Sabnis

Dr. Debansu Sarkar

Dr. K K Sarkar

Dr. Debanga Sarma

Dr. Rajeev Sarpal

Dr. Rohit Sethi

Dr. Shah Utsav Shailesh

Dr. S D Shamsundar

Dr. Anuj Sharma

Dr. Amit Sharma

Dr. Dipankar Sharma

Dr. Gopal Sharma

Dr. Ashish Kumar Sharma

Dr. Neeraj Kumar Sharma

Dr. Varun Sharma

Dr. Gurpremjit Singh

Dr. Moses Arun Singh

Dr. Satyajeet Kr Singh

Dr. Sekharjit Singh

Dr. Somorendo Singh

Dr. Sunil Singhal

Dr. Suraj Sochuk

Dr. R Srirathran

Dr. Arnulf Stenzl

Dr. K Subramaniyan

Dr. C Subramayan

Dr. Sanjay Sureka

Dr. Rajeev T P

Dr. Rajesh Taneja

Dr. Punit Tiwari

Dr. Jyoti Mohan Tosh

Dr. Sambit Tripathi

Dr. Rohit Upadhyay

Dr. Venugopalan A V

Dr. Shanmugha Das K V

Dr. Saurabh Vashishtya

Dr. Venkatesh

Dr. Vivek Venkatramani

Dr. Waheedu Zzaman
